# Landscape of Noncoding RNA in the Hypoxic Tumor Microenvironment

**DOI:** 10.3390/genes16020140

**Published:** 2025-01-24

**Authors:** Lianfeng Gong, Chuanxin Zou, Haixia Zhang, Fei Yang, Gui Qi, Zhaowu Ma

**Affiliations:** School of Basic Medicine, Health Science Center, Yangtze University, Jingzhou 434023, China; 2022710991@yangtzeu.edu.cn (L.G.); zouchuanxin8@163.com (C.Z.); 2022711003@yangtezu.edu.cn (H.Z.); finnyang@yangtzeu.edu.cn (F.Y.); qigui18@yangtzeu.edu.cn (G.Q.)

**Keywords:** long noncoding RNAs, circular RNAs, hypoxic microenvironment, cancer progression, clinical implications

## Abstract

Amidst the prevalent and notable characteristic of a hypoxic microenvironment present in the majority of solid tumors, a burgeoning number of studies have revealed the significance of noncoding RNAs (ncRNAs) in hypoxic tumor regions. The transcriptome of cancers is highly heterogeneous, with noncoding transcripts playing crucial roles. Long noncoding RNAs (lncRNAs) and circular RNAs (circRNAs) are two distinctive classes of ncRNA that are garnering increasing attention. Biologically, they possess intriguing properties and possess significant regulatory functions. Clinically, they present as promising biomarkers and therapeutic targets. Additionally, recent research has evaluated the clinical applications of these ncRNAs in RNA-based treatments and noninvasive liquid biopsies. This review provides a comprehensive summary of recent studies on lncRNAs and circRNAs within the hypoxic tumor microenvironment. Furthermore, the clinical significance of lncRNAs and circRNAs in cancer diagnosis and treatment is emphasized, which could pave the way for the development of effective targeted therapies.

## 1. Introduction

The tumor microenvironment (TME) is composed of various components, including the blood vessels, extracellular matrix, and stromal cells (e.g., endothelial cells, fibroblasts, immune cells, and mesenchymal stem cells), as well as secreted factors, like growth factors and cytokines [1]. Hypoxia constitutes a notable feature within the microenvironment of solid tumors [2]. Notably, hundreds or even thousands of coding and noncoding genes participate in complex adaptive mechanisms through which rapidly growing tumor cells adapt to hypoxia [3,4].

Noncoding RNAs (ncRNAs) mainly include microRNAs (miRNAs), long ncRNAs (lncRNAs), circular RNAs (circRNAs), and small nucleolar RNAs (snoRNAs) [5]. To date, the human database project has estimated the presence of 2654 miRNAs, 96,411 lncRNAs, 768,986 circRNAs, and 2064 snoRNAs [6,7,8,9]. Their presence can be readily detected in bodily fluids, such as tissues, serum, plasma, urine, and saliva [10]. Advancements in high-throughput technology and extensive research have shown that dysregulated ncRNAs participate in tumor initiation and metastasis through diverse molecular pathways [11,12,13]. In recent years, the clinical significance of ncRNAs in cancer has attracted considerable attention.

Although numerous protein-coding genes regulated by HIFs (hypoxia-inducible factors) contribute to the adaptation of tumor cells to hypoxia, the response of ncRNAs to hypoxia and their role in hypoxia-related cancer remain largely elusive. Recent developments in miRNAs in the context of hypoxia have been summarized in a previous review [14,15,16]. However, the role and mechanisms of hypoxia-related lncRNAs and circRNAs in cancer development have not been systematically investigated. In this review, we summarize the regulatory mechanisms exhibited by lncRNAs and circRNAs in their interaction with hypoxia and highlight their clinical significance in the diagnosis and treatment of cancer.

## 2. LncRNAs and circRNAs Act as Novel Players in the Hypoxic Tumor Microenvironment

Among the ncRNA subcategories, lncRNAs and circRNAs are recognized as essential regulators by diverse mechanisms of action in various pathophysiological processes [17,18,19]. LncRNAs, a diverse and extensive class of regulatory transcripts of at least 200 nucleotides in length, are generated from various genomic regions, encompassing promoter sequences, exons, antisense strands, enhancer elements, untranslated regions (UTRs) at both the 3′ and 5′ ends, and introns, as well as intergenic and intragenic segments [19,20,21]. However, certain scholars contend that a size cutoff of 200 nucleotides serves as a practical threshold in biochemical and biophysical RNA purification procedures, depleting the majority of infrastructural RNAs. Consequently, it is recommended to delineate ncRNAs exceeding 500 nucleotides as lncRNAs [22]. CircRNAs, a subtype of endogenous ncRNAs, are generated via an alternative splicing process known as backsplicing, exhibiting reduced susceptibility to exonuclease degradation due to their resultant circular molecules being devoid of terminal 5′ caps and 3′ polyadenylated tails, thereby conferring upon them enhanced stability relative to their linear RNA counterparts [23,24,25].

LncRNAs and circRNAs regulate gene expression through multiple mechanisms, including epigenetic recruitment, transcription regulation, post-transcriptional modulation, RNA/protein modifications, and encoding functional peptides [26,27,28,29,30,31]. The most prevalent mechanism among them involves lncRNAs and circRNAs functioning as competing endogenous RNAs (ceRNAs) or natural miRNA sponges, which engage in communication and co-regulation through competitive binding to shared miRNAs. A comprehensive understanding of this novel RNA crosstalk will provide profound insights into gene regulatory networks and have profound implications for cancers [32,33]. Additionally, they are attracting considerable attention owing to their substantial abundance, specific expression patterns, crucial functional roles in various diseases, and promising avenues for clinical application [28,34,35,36,37].

**Figure 1:**
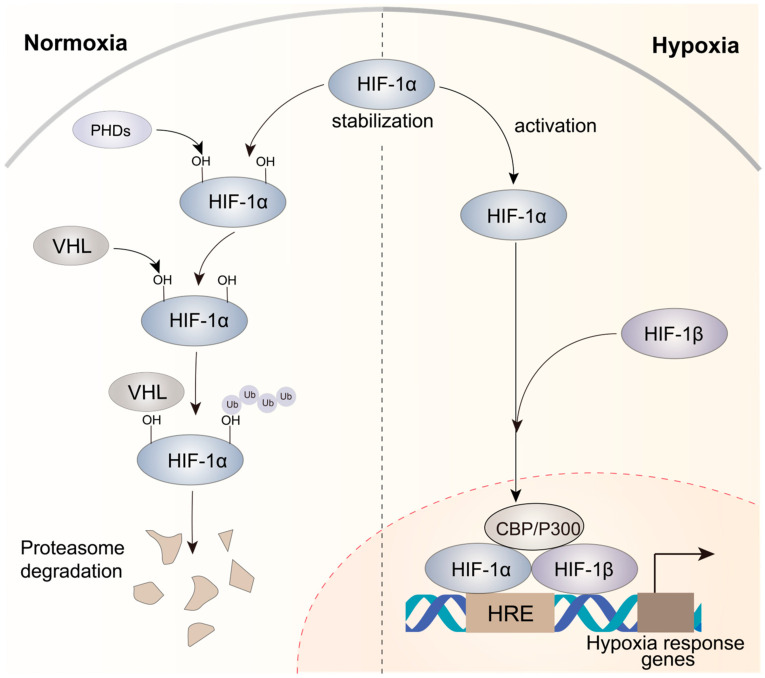
Mechanisms of HIF-1α activation under normoxic and hypoxic conditions in cancer. Under normoxic circumstances, HIF-1α displays instability, while under hypoxic conditions, it attains stability. The HIF-1α–HIF-1β heterodimer is recruited to HREs, activating the expression of hypoxia response genes that contribute to complex processes involved in tumor progression.

The microenvironment of solid tumors often exhibits hypoxia, which results from unrestrained and rapid tumor cell growth that limits the availability of oxygen [38]. HIFs are crucial for hypoxic responses [39], and increased HIF levels are frequently linked to a poor prognosis in various tumor types [40,41]. HIFs are heterodimeric proteins comprising a constantly expressed subunit (HIF-1β) and an oxygen-sensitive subunit (HIF-1α, HIF-2α, or HIF-3α). Under normal oxygen conditions, the HIF-1α subunit undergoes hydroxylation by dioxygenases called PHD1, PHD2, and PHD3. Subsequently, the hydroxylated prolines (PHDs) are recognized by the von Hippel–Lindau (VHL) E3 ubiquitin ligase, resulting in the ubiquitination of HIF-1α (Figure 1). However, under hypoxic conditions, PHDs cannot use oxygen as their co-substrate, resulting in the inhibition of hydroxylation. This inhibition leads to the accumulation of non-hydroxylated HIF-α subunits, which eventually translocate to the nucleus (Figure 1). In the nucleus, a heterodimeric complex consisting of HIF-1α and HIF-1β binds to hypoxia-responsive elements (HREs) on the promoters of hypoxia-responsive genes and drives the transcriptional activation of target genes after the recruitment of CBP/P300, a co-activator [42,43]. This process initiates a sequential cascade of cellular adaptations to hypoxia, encompassing amplified cell proliferation and angiogenesis, diminished apoptosis, and increased invasion and metastasis.

LncRNAs and circRNAs modulated by hypoxia have been shown to exert substantial effects on the hypoxic TME, including tumor metabolism, immune escape, angiogenesis, migration/invasion, apoptosis, and growth/proliferation [42,44]. Furthermore, hypoxia signaling can be regulated by lncRNAs and circRNAs through distinct mechanisms, particularly through the stabilization of HIF-1α [45,46]. However, there are still a large number of lncRNAs and circRNAs that have not been identified as regulatory factors for hypoxia signaling. Consequently, the identification and analysis of novel hypoxia-related lncRNAs and circRNAs, specifically those undergoing expression alterations in response to hypoxic conditions or interacting with HIFs, may offer valuable perspectives for the diagnosis and treatment of cancer and promote the development of novel treatments based on ncRNA-targeted strategies.

## 3. Hypoxia-Related lncRNAs in Cancers

**Figure 2:**
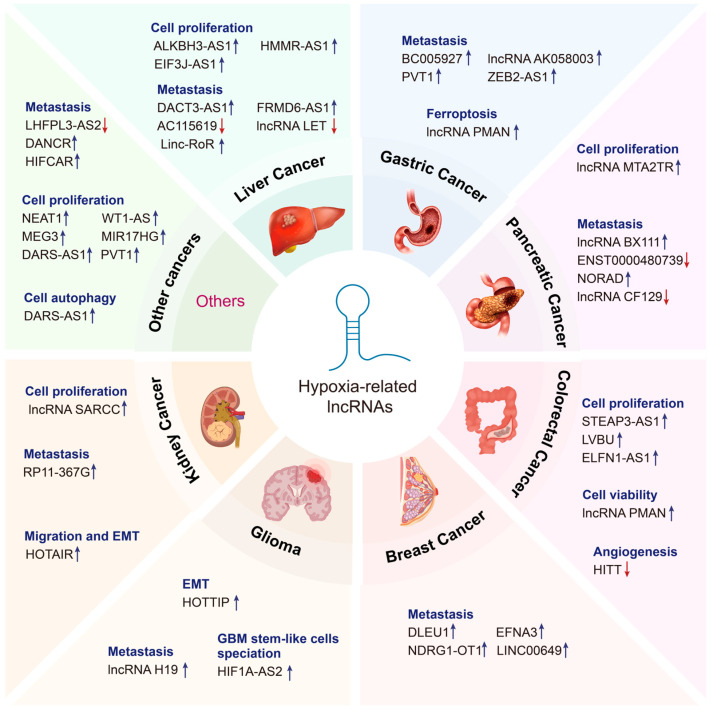
Emerging roles of hypoxia-related lncRNAs in cancer. Hypoxia-related lncRNAs perform diverse functions in various cancer types, including liver cancer, gastric cancer, pancreatic cancer, colorectal cancer, breast cancer, glioma, kidney cancer, and so on. Upregulated lncRNAs are indicated by the blue arrow, whereas downregulated lncRNAs are indicated by the red arrow.

In the context of oxygen deprivation, dysregulation of hypoxia-associated lncRNAs facilitates the adaptation of tumor cells to microenvironmental stress, leading to enhanced aggressiveness, immune evasion, invasive angiogenesis, and glycolysis. These effects may occur in a HIF-dependent or HIF-independent manner. In the following sections, we provide a comprehensive summary of the functions and mechanisms of lncRNAs that interact with hypoxia in cancer (Figure 2 and Table 1).

### 3.1. Gastrointestinal Cancers

#### 3.1.1. Liver Cancer

Hepatocellular carcinoma (HCC) is the most prevalent type of primary liver cancer and is the most common cause of cancer-related death [91]. Multiple lines of studies have revealed that targeting hypoxia-related lncRNAs may represent a novel strategy for HCC. These lncRNAs can influence the stability or functionality of their partners through their interactions with specific proteins, thereby modulating the biological behavior of HCC cells. For instance, the lncRNA DACT3-AS1 has been shown to promote metastasis in HCC. Mechanistically, DACT3-AS1 increases the deacetylation of FOXA3 by facilitating its interaction with HDAC2, leading to a decrease in FOXA3 protein levels. Furthermore, by binding to the pyruvate kinase M2 (PKM2) promoter area, the transcription factor FOXA3 might prevent PKM2, an enzyme that plays an important role in promoting HCC invasiveness, from being expressed [47]. Beyond their interactions with specific proteins, hypoxia-related lncRNAs also participate in tumorigenesis and progression by modulating the degradation or accumulation of mRNAs. The lncRNA-LET facilitates hypoxia-induced metastasis in HCC by modulating the accumulation and stability of HIF-1α mRNA. This modulation occurs through the downregulation of nuclear factor 90 (NF90) protein via the ubiquitin–proteasome pathway [50]. Similarly, FRMD6-AS1 was found to increase SENP1 protease activity, thereby increasing the stability of HIF-1α under hypoxia [51]. HIF-1α-induced ALKBH3-AS1 played a tumorigenic role by enhancing the stability of ALKBH3 mRNA. ALKBH3 is an oncogene that promotes the invasion and proliferation of HCC cells [48]. A few studies have shown that some hypoxia-related lncRNAs contain short ORFs (sORFs, short open reading frames, length < 300 nt) and are capable of encoding a small peptide with crucial biological functions in hypoxia. For instance, the hypoxia-responsive lncRNA AC115619 inhibits the growth of HCC cells through its peptide-coding functions. Mechanistically, through its interaction with WTAP, the encoded micropeptide AC115619–22aa prevents the m6A methyltransferase complex from forming. This interaction effectively reduces the increase in m6A modification, which is associated with high malignancy in tumors [49]. Hypoxia-related lncRNAs also function as sponges for miRNAs, thereby chestrating the intricate regulation of downstream gene expression and influencing the progression of HCC. Some lncRNAs (e.g., linc-RoR, EIF3J-AS1, and HMMR-AS1) can be induced by hypoxia and exert a vital role in hypoxia-induced cancer development by sponging miRNAs and influencing the expression of their targets [52,53,54]. These findings have illuminated the pivotal role of hypoxia-related lncRNAs in HCC through a myriad of mechanisms, primarily influencing gene expression and signaling pathway activity to promote HCC progression and metastasis.

#### 3.1.2. Gastric Cancer

Gastric cancer (GC) ranks among the top five most widespread types of cancer globally and constitutes the third principal cause of cancer-related mortality worldwide [92]. Studies have demonstrated that hypoxia-related lncRNAs can modulate GC progression by influencing the expression of downstream genes. For instance, HIF-1α-induced PMAN enhances ferroptosis resistance by facilitating the cytoplasmic translocation of ELAVL1 and enhancing SLC7A11 expression under hypoxic conditions, thereby favoring the proliferation and progression of GC cells [55]. Furthermore, DNA methylation stands as a crucial epigenetic modification, wielding a profound influence over the regulation of a myriad of oncogenes and tumor suppressor genes. Notably, hypoxia-related lncRNAs are deeply entwined in the regulation of this methylation process, underscoring their significance in the complex landscape of cancer biology. In hypoxic GC cells, the lncRNA BC005927 regulates the ephrin type B receptor 4 (EPHB4), a metastasis-related gene, to influence the metastatic and invasive abilities of GC cells. Knockdown of BC005927 reduces EPHB4 expression by modulating the DNA methylation of EPHB4 in GC cells [56]. Similarly, the lncRNA AK058003 is upregulated under hypoxic conditions and regulates the metastasis-associated gene SNCG via DNA demethylation to modulate the hypoxia-induced metastasis of GC [57]. Numerous investigations have illuminated the intricate role of hypoxia-related lncRNAs in the modulation of HIF-1α expression via miRNA sponges, thereby influencing GC progression. For example, the lncRNAs PVT1 (plasmacytoma variant translocation 1) and ZEB2-AS1 increase HIF-1α expression and contribute to GC cell growth and invasion by influencing miRNA targets [58,59]. It is commonly known that a promising treatment approach for cancer involves strategically targeting HIF-1α and HIF-2α. However, resistance to treatment is an inevitable aspect of cancer therapy [93]. The aforementioned studies indicate that hypoxia-related lncRNAs extensively modulate the progression of GC, offering a novel perspective for guiding GC therapy by targeting these lncRNAs.

#### 3.1.3. Pancreatic Cancer

The incidence and mortality of pancreatic cancer have been increasing steadily, while the rates of other prevalent cancers have been on the decline [94]. Numerous studies have found the potential clinical applications of hypoxia-related lncRNAs in pancreatic cancer. These lncRNAs are capable of regulating the expression level of oncogenes by recruiting transcription factors to their promoter regions. For example, lncRNA MTA2TR upregulated the transcription of metastasis-associated protein 2 (MTA2) by recruiting activating transcription factor 3 to the MTA2 promoter region in pancreatic cancer. MTA2 facilitates the stabilization of the HIF-1α protein through deacetylation [62]. Furthermore, the hypoxia-related lncRNAs emerge as pivotal factors, orchestrating the ubiquitin-dependent degradation of transcription factors, thus intricately modulating the expression and activity of target genes. The lncRNA CF129 can effectively suppress *FOXC2* transcription by inducing the MKRN1-mediated ubiquitin-dependent degradation of p53, thereby inhibiting cancer cell proliferation and invasion [60]. Hypoxia-related lncRNAs can also indirectly affect the expression level of HIF-1α. Downregulated ENST00000480739 suppresses HIF-1α expression by promoting the transcription of *OS-9*, thereby contributing to tumor progression in pancreatic cancer [64]. Additionally, a hypoxia-related lncRNA can function as a ceRNA that binds to a specific miRNA, thereby alleviating the inhibitory effect of that miRNA on its targets. Overexpression of NORAD increases cancer cell migration and invasion, whereas its knockdown prevents epithelial-to-mesenchymal transition (EMT) and metastasis. Mechanistically, NORAD modulates the expression of RhoA by acting as a ceRNA of miR-125a-3p [61].

#### 3.1.4. Colorectal Cancer

Accumulating research has found that hypoxia-related lncRNAs can modulate the advancement of colorectal cancer (CRC). Hypoxia-related lncRNAs also affect CRC development by influencing post-transcriptional processes such as alternative splicing and mRNA stabilization. A study showed that higher LUCAT1 expression indicated a worse prognosis in CRC. Mechanistically, the interaction between LUCAT1 and polypyrimidine tract-binding protein 1 (PTBP1) facilitates the binding of some DNA damage-related genes with PTBP1, consequently leading to changes in the alternative splicing of these genes [66]. Hypoxia-induced lncRNA STEAP3-AS1 activates the Wnt/β-catenin signaling pathway through the inhibition of m6A-mediated STEAP3 mRNA degradation, ultimately facilitating the progression of CRC [67]. In addition, hypoxia-related lncRNAs can also affect crucial signaling cascades to influence CRC progression by functioning as ceRNAs. For instance, HIF-1α-induced lncRNA LVBU, acting as a sponge of miR-10a/miR-34c, leads to the reprogramming of the urea cycle and the phenotype of polyamine synthesis by modulating BCL6 and p53, thereby facilitating CRC progression [68]. Similarly, ELFN1-AS1, as a downstream effector of hypoxia, promotes CRC cell proliferation and invasion by upregulating TRIM14 through miR-191-5p sponging [69]. Notably, hypoxia-related lncRNAs possess the capacity to regulate HIF-1α expression. For instance, the lncRNA HITT is significantly downregulated and directly binds to YB-1, hindering the interaction of the 5′-UTR of HIF-1α with YB-1 and inhibiting HIF-1α translation [65]. These investigations underscore the essential roles that hypoxia-related lncRNAs play in the modulation of oncogene expression and vital signaling pathways within CRC, ultimately contributing to the trajectory of cancer progression and prognostic outcomes. Thus, the strategic targeting of these hypoxia-related lncRNAs emerges as a promising therapeutic avenue.

### 3.2. Breast Cancer

Breast cancer (BC) ranks among the three most prevalent cancers globally. Early-stage BC is regarded as potentially treatable. Studies have revealed the potential of hypoxia-related lncRNAs as targets for the diagnosis and therapy of BC disease. Hypoxia-related lncRNAs emerge as pivotal players in the malignant behavior and progression of BC, intricately modulating HIF-1α and its downstream signaling pathways. For example, lncRNA DLEU1 is upregulated and promotes the malignant behavior of BC cells. Mechanistically, DLEU1 may serve as a cofactor and activate HIF-1α-mediated transcription of *CKAP2*, thereby activating the ERK and STAT3 signaling pathways to promote tumor growth [70]. Studies have confirmed that LINC00649 can influence cancer progression by affecting certain key signaling pathways [95,96]. Notably, LINC00649 is also upregulated and promotes BC progression and metastasis by increasing HIF-1α mRNA stability through interacting with the NF90/NF45 complex [71]. Other lncRNAs can be induced by hypoxic conditions, exerting a crucial influence on BC development through their competitive binding to miRNAs. A study revealed that the high expression of EFNA3 lncRNAs promoted the metastatic dissemination of BC cells. Functional assays revealed that under hypoxic conditions, EFNA3 suppressed the inhibitory effects of miR-210 on EFNA3 mRNA and facilitated efficient transcription, leading to the accumulation of Ephrin-A3 [72]. Similar to EFNA3, the transactivation of NDRG1-OT1 by HIF-1α can enhance cancer cell growth and migration by sponging miR-875-3p. Moreover, NDRG1-OT1 encodes a 66-amino acid peptide in the 29–226 bp region [73]. Thus, the intricate interplay between lncRNAs and hypoxia intricately contributes to the malignant behavior of BC cells at both transcriptional and post-transcriptional levels. These findings uncover the complex regulatory network governing hypoxia-related lncRNAs in BC progression and offer new potential targets and research avenues for BC treatment.

### 3.3. Glioma

Glioma is the most prevalent and fatal brain tumor in adults [97]. Consequently, novel and curative treatment approaches are urgently required. Hypoxia-related lncRNAs engage in intricate interactions with RNA-binding protein complexes, playing a pivotal role in the adaptive and malignant behaviors of glioma cells by stimulating the expression of oncogenes. A study showed that HIF1A-AS2 facilitates the specialization and hypoxic adaptation of glioma stem cells in the TME. Mechanistically, the direct interaction between HIF1A-AS2 and the mRNA-binding complexes of DHX9 and IGF2BP2 stimulates the expression of HMGA1, which possesses oncogenic properties [74]. Additionally, hypoxia-related lncRNAs also function as ceRNAs to promote glioma progression. Overexpression of the lncRNA H19 can increase the expression of β-catenin by acting as a ceRNA of miR-181d [75]. Similarly, hypoxia-induced upregulation of HOTTIP can promote metastasis in gliomas. Mechanistically, HOTTIP functions as a sponge of miR-101, inhibiting its endogenous activity and subsequently enhancing Zinc Finger E-Box Binding Homeobox 1 (ZEB1) expression to promote EMT [76]. These results support the notion that hypoxia-related lncRNAs facilitate the specialization, hypoxic adaptation, and EMT of glioma cells, ultimately contributing to tumor progression. Consequently, hypoxia-related lncRNAs emerge as promising novel therapeutic targets for patients grappling with glioma.

### 3.4. Renal Cell Carcinoma

Accumulating studies have found that hypoxia-related lncRNAs can regulate renal cell carcinoma (RCC) progression. These lncRNAs engage in intricate interactions with nuclear proteins, thereby influencing their stability and functional dynamics. Such interactions, in turn, intricately modulate downstream signaling pathways, ultimately impacting the biological behavior of tumor cells. For example, LncRNA-SARCC inhibits hypoxic cell cycle progression in RCC cells harboring VHL mutations, yet it reverses this inhibition in RCC cells with restored VHL function. Mechanistic analysis demonstrates that LncRNA-SARCC post-transcriptionally modulates the androgen receptor (AR) by physically associating with and destabilizing the AR protein, subsequently suppressing the AR/HIF-2α/C-MYC signaling pathway. Conversely, HIF-2α transcriptionally regulates the expression of *LncRNA-SARCC* by binding to hypoxia-responsive elements located on the *LncRNA-SARCC* promoter [77]. A study showed that the RP11-367G18.1 variant 2 played an indispensable role in promoting the hypoxia-induced development and metastasis of RCC. Mechanistically, the RP11-367G18.1 variant 2 regulates the expression of hypoxia-related genes by interacting with the histone acetyltransferase p300 to promote acetylation of histone 4 at lysine 16 (H4K16Ac) [78]. Besides interacting with proteins, hypoxia-related lncRNAs can also function as sponges for miRNAs, indirectly regulating downstream signaling pathways and further impacting the trajectory of RCC progression. A study demonstrated a positive correlation between increased HOTAIR levels and tumor progression. Functional experiments indicated that HOTAIR was sponging miR-217, promoting RCC progression partially through the HIF-1α/AXL signaling pathway [79]. Notably, a recent study has also shown HOTAIR modulates several target genes through epigenetic mechanisms and regulates diverse oncogenic cellular and signaling pathways [98].

### 3.5. Other Cancers

Increasing numbers of research studies have also demonstrated that hypoxia-related lncRNAs can modulate the progression of other cancers through the mechanisms described above, primarily through their modulation of the HIF-α signaling pathway. In cervical cancer, lincRNA-p21, a lncRNA that responds to hypoxia, plays a crucial role in enhancing glycolysis under hypoxic conditions. Hypoxia/HIF-1α-induced lincRNA-p21 disrupts the protein–protein interaction between VHL and HIF-1α by binding to both factors, thereby inhibiting the VHL-mediated ubiquitination of HIF-1α and leading to the accumulation of HIF-1α [86]. Additionally, studies have also reported that hypoxia-related lncRNAs such as PVT1, DANCR, and HIFCAR facilitate the stabilization and transcriptional network of HIF-1α to facilitate cancer progression [80,81,85]. Several other hypoxia-related lncRNAs have emerged as promising candidates for diagnostic and prognostic biomarkers across a spectrum of cancers, functioning as miRNA sponges or chromatin scaffolds that facilitate protein interactions [82,83,84,99]. Furthermore, WT1-AS and MEG3 have been shown to modulate tumor growth through their association with histone methylation. Beyond histone methylation, hypoxia-related DARS-AS1 also regulates protein stability through m6A modification and ubiquitination [87,88]. In summary, these mechanisms mediated by hypoxia-related lncRNAs ultimately influence oncogenic signaling pathways and tumor behavior, thereby unveiling promising avenues for potential therapeutic interventions.

## 4. Hypoxia-Related circRNAs in Cancers

**Figure 3:**
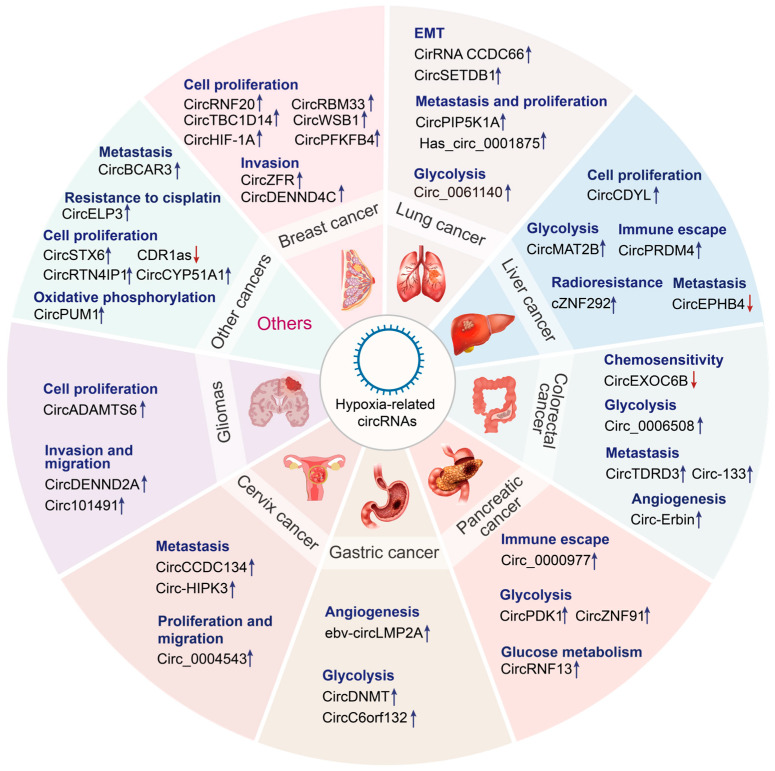
Emerging roles of hypoxia-related circRNAs in cancers. Hypoxia-related circRNAs perform diverse functions in various cancer types, including lung cancer, liver cancer, colorectal cancer, pancreatic cancer, gastric cancer, cervical cancer, glioma, and so on. Upregulated circRNAs are indicated by the blue arrow, whereas downregulated circRNAs are indicated by the red arrow.

CircRNAs are crucial for the regulation of genes, and their dysregulation may promote tumorigenesis [100]. Recent studies on RNA molecules have revealed the complex mechanisms underlying the expression of cancer-related genes [101]. Tumor stem cell maintenance, vascularization, invasion, metastasis, energy metabolism, cell immortalization, genetic instability, and chemotherapy resistance are influenced by hypoxic TME and circRNA functions [102]. To date, the relationship among circRNAs, hypoxia/HIFs, and cancer has been examined only in preliminary studies. Therefore, in-depth investigation is necessary to discover new biomarkers and targets for cancer diagnosis and treatment. The following sections discuss the latest research results about the function of circRNAs that interact with hypoxia in various malignancies (Figure 3 and Table 2).

### 4.1. Gastrointestinal Cancers

#### 4.1.1. Liver Cancer

HCC is a prevalent cancer, posing a significant challenge to global health care [146]. Research has confirmed that hypoxia-related circRNAs may have a role in the development and advancement of liver cancer. Hypoxia-related circRNAs often function as miRNA sponges to derepress their target genes. For example, upregulated circMAT2B increases the expression of its downstream gene PKM2 by sequestering miR-338-3p in HCC. PKM2 is a crucial enzyme contributing to glycolysis and HCC progression [116]. Certain hypoxia-related circRNAs regulate the immune microenvironment of HCC, influencing the infiltration and activity of immune cells. A study showed that circPRDM4 facilitated the immune evasion of HCC cells by promoting the recruitment of HIF-1α to the promoter region of the CD274 gene, which encodes programmed death ligand 1, consequently suppressing the infiltration of CD8+ T cells in the hypoxic TME [117].

Hypoxia-related circRNAs have been reported to modulate the onset and progression of HCC by regulating the Wnt/β-catenin and phosphatidylinositol 3-kinase (PI3K) signaling pathways. For instance, hypoxia-induced cZNF292 promotes HCC development in a time-dependent manner. Downregulation of cZNF292 promotes the nuclear translocation of SOX9 and concomitantly suppresses the Wnt/β-catenin pathway, consequently attenuating the proliferation and radiosensitivity of hypoxic hepatocytes [118]. Circ-CDYL is overexpressed in HCC and functions as a ceRNA by interacting with mRNAs encoding HIF-1AN and HDGF, thereby sequestering miR-328-3p and miR-892a, respectively. This phenomenon results in the PI3K-threonine kinase (AKT)—mechanistic target of rapamycin complex 1 (mTORC1)/β-catenin and NOTCH2 signaling pathways—being activated, triggering the expression of important effector molecules, such as BIRC5 and MYC proto-oncogene, which consequently promote the self-renewal of HCC cells [119]. Conversely, circ-EPHB4 downregulation inhibits the growth of HCC by regulating the PI3K-AKT pathway and HIF-1α [120]. Therefore, an increasing amount of research demonstrates that hypoxia-related circRNAs are crucial players in HCC progression. The strategic targeting of these hypoxia-related circRNAs presents a promising avenue for therapeutic intervention in HCC.

#### 4.1.2. Colorectal Cancer

The CRC ranks as the second primary contributor to cancer-related mortality globally and the third most prevalent malignancy [147]. Increasing numbers of research studies have also revealed that hypoxia-related lncRNAs can modulate the progression of colorectal cancers. A fundamental mechanism through which hypoxia-related circRNAs exert their influence on CRC progression lies in the modulation of HIF-1α expression. A study demonstrated that circTDRD3 enhanced the malignant progression of CRC by absorbing miR-1231 to increase HIF-1α expression [121]. Li et al. found that clinical samples from patients with CRC exhibited increased expression of circ-ERBIN, which showed a positive correlation with aggressive phenotypes. Functional assays revealed that circ-Erbin facilitated cap-independent translation of HIF-1α by sequestering miR-138-5p and miR-125a-5p, which collectively regulated the eukaryotic translation initiation factor 4EBP-1 [123]. Another study showed that circ_0006508 is remarkably upregulated in CRC and is related to the TNM stage and overall survival. Mechanistically, HIF-1α-induced circ_0006508 directly sequesters miR-1272, suppressing glycolysis [122]. Hypoxia-related circRNAs also modulate key signaling pathways involved in CRC cell growth, survival, and metastasis. CRC tissues exhibit decreased expression of circEXOC6B, which is correlated with an unfavorable prognosis. Mechanistically, circEXOC6B binds to RRAGB and suppresses its heterodimer formation, thereby inhibiting the mTORC1 pathway and decreasing HIF-1α expression. Additionally, HIF-1α interacts with a specific region in the RRAGB promoter to increase its transcription [124]. Circ-133 is upregulated in plasma exosomes from patients with CRC, and its expression increases with disease progression. Hypoxia-induced exosomal circ-133 is transferred to normoxic cells, leading to the promotion of cancer metastasis through modulation of the miR-133a/GEF-H1/RhoA axis. However, knockdown of circ-133 has been shown to prevent CRC metastasis in animal models [125].

#### 4.1.3. Pancreatic Cancer

Hypoxia-related circRNAs have also been shown in a growing body of research to modulate the progression of pancreatic cancer. Hypoxia-related circRNAs often function as miRNA sponges to regulate the expression of HIF-1α. Research revealed that circ_0000977 promoted immune responses in pancreatic cancer under hypoxic settings. Upregulated circ_0000977 increased the expression of ADAM10 and HIF-1α by competitively binding to miR-153, leading to the suppression of natural killer cells in the hypoxic TME [126]. Meanwhile, HIF-1α plays a pivotal role in modulating the biogenesis of these circRNAs, thereby impacting tumor growth and metastasis through their intricate interplay with miRNAs. A study showed that the biogenesis of circRNF13 was mediated by HIF-1α and EIF4A3. Functional analysis revealed that circRNF13 promoted glucose metabolism and accelerated the development of pancreatic cancer. Mechanistically, circRNF13 regulates the miR-654-3p/PDK3 pathway to facilitate tumor formation and metastasis in pancreatic cancer [127]. Similarly, circPDK1 is upregulated in both tumor tissues and serum exosomes in pancreatic cancer and is correlated with an unfavorable prognosis. CircPDK1 is activated by HIF-1α through transcriptional regulation and serves as a ceRNA of miR-628-3p, leading to the activation of the BPTF/c-myc axis. Furthermore, circPDK1 functions as a scaffold to enhance the interaction between 65 kDa Myc box-dependent interacting protein 1 (BIN1), a tumor suppressor that interacts with c-myc to limit its transcriptional activity, and UBE2O, thereby facilitating BIN1 degradation by UBE2O [128]. CircZNF91 has been demonstrated to promote gemcitabine (GEM) resistance. Mechanistically, HIF-1α-induced exosomal circZNF91 is transferred to normoxic pancreatic cancer cells and serves as a ceRNA of miR-23b-3p, thereby relieving the inhibitory effects of miR-23b-3p on Sirtuin1 (SIRT1) expression. Consequently, upregulated SIRT1 enhances HIF-1α stability through deacetylation, resulting in glycolysis and GEM resistance in recipient pancreatic cancer cells [129]. Collectively, these studies revealed the intricate interplay between hypoxia-related circRNAs and HIF-1α, accelerating the deterioration process and influencing the therapeutic response in pancreatic cancer through a myriad of mechanisms.

#### 4.1.4. Gastric Cancer

In developing nations, GC is a major cause of cancer-related mortality. Hypoxia-related circRNAs are considered novel and promising therapeutic targets for GC [147]. Ebv-circLMP2A expression is positively related to microvessel density and the expression of HIF-1α and VEGFA in EBV-associated GC. HIF-1α-induced ebv-circLMP2A facilitates angiogenesis through the KHSRP/VHL/HIF-1α/VEGFA axis under hypoxic conditions [130]. Additionally, hypoxia-related circRNAs regulate the expression of key oncogenes by acting as a ceRNA, which in turn influences the malignant progression of tumors. A study showed that circDNMT1 knockdown attenuated the malignant progression of GC cells. The oncogenic role of circDNMT1 is attributed to its ability to sequester miR-576-3p, which binds to the 3′-untranslated region of HIF-1α mRNA and adversely regulates it. CircDNMT1 modulates the miR-576-3p/HIF-1α axis, resulting in malignant progression and metabolic reprogramming in GC both in vivo and in vitro [131]. Under hypoxic conditions, circC6orf132 facilitates the progression and glycolytic activity of GC cells by sequestering miR-873-5p, resulting in increased expression of PRKAA1. PRKAA1 constitutes a critical subunit of the 5′-AMP-activated protein kinase (AMPK), which plays a pivotal role in promoting energy metabolism and tumor progression in GC [132].

#### 4.1.5. Other Gastrointestinal Cancers

In addition to the tumor types described above, hypoxia-related circRNAs also have a significant impact on other gastrointestinal cancers. Hypoxia-related circRNAs emerge as pivotal regulators of cancer progression, functioning as miRNA sponges that intricately modulate the expression of critical downstream oncogenes. In esophageal cancer, the splicing factor QKI promoted the generation of circBCAR3, which in turn facilitated tumorigenesis. This effect was achieved upon the binding of circBCAR3 to miR-27a-3p, which resulted in the upregulation of transportin-1 (TNPO1). The upregulation of TNPO1, in turn, enhances the proliferation, migration, and invasion of esophageal cancer cells [139]. CircSTX6 regulates the expression of non-muscle myosin heavy chain 9 (MYH9) via the circSTX6/miR-449b-5p and circSTX6/CUL2/HIF-1α pathways, promoting the proliferation and migration of pancreatic ductal adenocarcinoma (PDAC) cells [143]. Similarly, Huang et al. showed that circRTN4IP1 knockdown in intrahepatic cholangiocarcinoma (ICC) cells suppressed cell proliferation and glucose metabolism, whereas cell apoptosis was promoted by the miR-541-5p/HIF-1α axis [145]. Certain circRNAs promote energy metabolism and viability of cancer cells by maintaining mitochondrial stability and function. In esophageal squamous cell carcinoma (ESCC), circPUM1 promoted the progression of ESCC by inhibiting cancer cell pyroptosis. Mechanistically, circPUM1 maintains the stability of mitochondrial complex III, thereby enhancing oxidative phosphorylation and subsequent ATP production in ESCC cells. Additionally, ESCC cells use circPUM1 for cell adaptation [140]. In summary, hypoxia-related circRNAs, which regulate biological processes such as proliferation, migration, metabolism, and apoptosis of cancer cells, may serve as potential prognostic markers and therapeutic targets for patients with gastrointestinal cancers.

### 4.2. Breast Cancer

BC has the highest global incidence rate for women [147]. Despite recent advances in the early diagnosis and treatment of BC, a large number of patients with BC develop metastasis. Therefore, identifying novel biomarkers is necessary to provide more effective diagnostic and therapeutic methods for BC. CircRNAs intricately enhance the expression of HIF-1α through the competitive binding of specific miRNAs, consequently facilitating BC cell growth and metastasis. For example, circRNF20 is upregulated in BC and facilitates cancer cell proliferation and the Warburg effect. Mechanistically, the competitive binding of circRNF20 to miR-487a upregulates the expression of HIF-1α and facilitates the transcription of hexokinase II (HK2), a key enzyme that enhances glycolysis [103]. The circZFR has been shown to promote the progression of BC. Silencing of circZFR suppresses the viability, invasiveness, migration, colony-forming ability, and glycolytic activity of BC cells through the miR-578/HIF-1α axis [104]. CircRBM33 is upregulated in BC, and its silencing suppresses miR-542-3p-mediated expression of HIF-1α, subsequently inhibiting glycolysis and proliferation and enhancing apoptosis in BC cells [105]. In addition, circDENND4C and circTBC1D14 can also facilitate the development of BC under hypoxic conditions [106,107,148].

Recent studies have reported that hypoxia-related circRNAs can promote BC progression by decreasing the expression of the cell cycle inhibitors p53, p21, and p27. The hypoxia-inducible circWSB1 is upregulated in BC and promotes cancer cell proliferation. Mechanistically, the interaction between circWSB1 and ubiquitin-specific peptidase 10 (USP10) attenuates the USP10-mediated stabilization of p53 [108]. CircHIF-1α (hsa_circ_0004623) has been shown to facilitate cancer cell proliferation and metastasis in BC. Mechanistically, circHIF1A modulates nuclear factor IB (NFIB) expression and translocation via post-transcriptional and post-translational modifications, thereby leading to the AKT/STAT3 signaling pathway being activated and p21 being suppressed [109]. Additionally, a poor prognosis is linked to elevated circPFKFB4 expression. Mechanistically, hypoxia-induced circPFKFB4 directly interacts with both DDB1 and DDB2, facilitating the assembly of the CRL4DDB2 E3 ubiquitin ligase, which in turn mediates the ubiquitination of p27 [110].

### 4.3. Lung Cancer

Lung cancer is the primary cause of cancer-related mortality worldwide, and its most common subtype is non-small cell lung cancer (NSCLC) [149]. Hypoxia-related circRNAs exert intricate regulatory influence on lung cancer cell proliferation, metastasis, and cellular morphology via miRNA sponges. Circ-0001875 is markedly increased in NSCLC cell lines and tissues. It not only facilitates NSCLC cell proliferation and metastasis but also triggers filopodia extension in NSCLC cells. Mechanistically, circ-0001875 modulates SP1 expression by sponging miR-31-5p, thereby influencing EMT through the TGFβ/Smad2 signaling pathway. The negative regulation of circ-0001875 is mediated by SP1 through an AluSq-dependent feedback loop. However, this regulation is disrupted under hypoxic conditions owing to the competitive binding of HIF-1α to SP1 [111]. Similarly, circPIP5K1A is upregulated in NSCLC cells and modulates cancer cell proliferation and metastasis by binding to miR-600 and activating HIF-1α [112].

Lung adenocarcinoma (LUAD) is the most prevalent form of non-small cell lung cancer, which comprises around 40% of all lung cancer [150]. LUAD cells exhibit increased expression of circ-0000211, which promotes cancer cell migration through the miR-622/HIF-1α axis [113]. Similarly, upregulated circ-0061140 has been associated with a decreased survival rate in patients with LUAD. Circ-0061140 acts as a sponge of miR-653 to regulate the expression of HK2, thereby promoting the hypoxia-induced glycolytic activity, migration, and invasiveness of LUAD cells [114]. The expression of hypoxia-related circRNAs not only affects the migration of LUAD cells but also correlates closely with LUAD cell adaptation and drug resistance. The increased expression of CCDC66 in LUAD is attributed to the negative modulation of nAchR7α and the positive modulation of c-Met and FAK. In response to hypoxia, cells quickly upregulate phosphorylated c-Met, EMT, and SAE2, which consequently enhance drug resistance and EMT in LUAD [115]. Therefore, identifying hypoxia-related circRNAs that are potentially targetable in lung cancer progression is necessary to improve treatment efficacy.

### 4.4. Cervical Cancer

The second most frequent cause of cancer death for women between the ages of 20 and 39 is cervical cancer. Hypoxia-related circRNAs have been demonstrated to be crucial in regulating the progress of cervical cancer by modulating HIF-1α expression under hypoxic conditions [151]. For example, circCCDC134 is significantly upregulated in cervical cancer and facilitates tumor growth and metastasis. Mechanistically, circCCDC134 mediates nuclear recruitment of p65 and functions as a sponge of miR-503-5p to modulate myeloblastosis (MYB) expression, which is a transcription factor, in the cytoplasm, thereby facilitating HIF-1α transcription [133]. Upregulated circ_0004543 facilitated the viability, proliferation, migration, and invasiveness of cervical cancer cells while suppressing their apoptosis. These effects are mediated through the sequestration of hsa-miR-217 by circ_0004543, which increases HIF-1α levels in CC cells [134]. Similarly, upregulated circ-HIPK3 in cervical cancer cells sequesters miR-338-3p to increase the expression of HIF-1α, consequently enhancing the proliferation, colony formation, migration, and invasiveness of cervical cancer cells while suppressing their apoptosis [135]. In cervical cancer, circCCDC134, circ_0004543, and circ-HIPK3 all upregulate the expression of HIF-1α by acting as miRNA sponges, thus promoting the proliferation, migration, and invasion of cervical cancer cells and thereby accelerating tumor growth and metastasis.

### 4.5. Glioma

Gliomas, which originate from progenitor or neuroglial stem cells, are predominant tumors in the central nervous system. Therefore, identifying novel and effective biomarkers for the diagnosis and treatment of glioma is necessary [152]. Hypoxia-related circRNAs have the ability to function as miRNA sponges to regulate the expression of downstream genes. Circ101491 is found to be upregulated in both tumor tissues and peripheral plasma of glioma patients. Upregulated circ101491 is strongly linked to a poor prognosis in glioma. Hypoxia-induced upregulation of circ101491 can promote the invasiveness, metastasis, and viability of neighboring or residual glioma cells through exosomes. Mechanistically, circ101491 mediates the upregulation of the downstream target gene endothelin-1 (EDN1) by acting as a ceRNA of miR-125b-5p. Notably, high expression of EDN1 promotes the viability and invasion of glioma cells [138]. Similarly, another study showed that hypoxia-induced upregulation of circDENND2A promoted the invasiveness and migration of gliomas by sequestering miR-625-5p [136]. In addition to acting as miRNA sponges, circRNAs promote glioma cell proliferation and inhibit apoptosis through other mechanisms. CircADAMTS6 can drive the malignant progression of glioblastoma by suppressing apoptosis and facilitating cancer cell proliferation. Mechanistically, hypoxia-induced upregulation of circADAMTS6 is mediated by the transcription factor AP-1 and the RNA-binding protein TDP43 in glioblastoma. CircADAMTS6 promotes the progression of glioblastoma by facilitating the recruitment and stabilization of annexin A2 (ANXA2) through a proteasome-dependent mechanism. ANXA2 exerts its effects by inhibiting apoptosis and promoting cell proliferation and self-renewal of stem cells [137]. Consequently, hypoxia-related circRNAs may serve as potential prognostic markers and therapeutic targets for patients with glioma.

### 4.6. Other Cancers

Recent research has revealed that hypoxia-related circRNAs are dysregulated in most cancer types, thus playing an indispensable role in the development of cancer, either as tumor suppressors or as oncogenes [101]. For example, in bladder cancer, circELP3 is upregulated in the hypoxic microenvironment and is positively correlated with tumor development. Bladder cancer cells with reduced circELP3 expression have impaired self-renewal capacity. In addition, silencing of circELP3 enhances apoptosis and reduces cisplatin resistance in bladder cancer cells [141]. CircRNA CDR1as is downregulated in ovarian cancer and serves as a tumor suppressor. It sequesters miR-135b-5p, increasing the expression of HIF-1AN and suppressing the proliferation of ovarian cancer cells [144]. In hypoxic osteosarcoma cells, circCYP51A1 can regulate glycolysis, proliferation, migration, and invasion by modulating the miR-490-3p/KLF12 axis [142]. These studies revealed that hypoxia-related circRNAs exert pivotal roles in cancer by regulating tumor progression and even chemotherapy drug sensitivity. Their influence on cancer cell behavior underscores their importance as potential therapeutic targets in cancer treatment.

## 5. Clinical Relevance of Hypoxia-Related lncRNAs and circRNAs in Cancers

**Figure 4:**
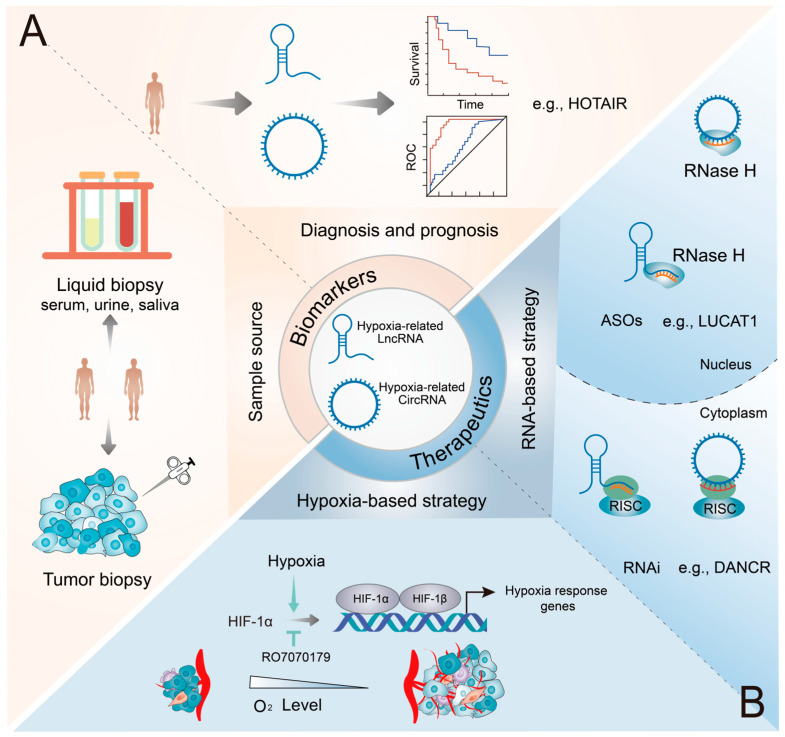
Potential applications of hypoxia-related lncRNAs and circRNAs as biomarkers and therapeutic targets in cancers. (**A**) Diagnostic potential of hypoxic ncRNAs. These ncRNAs can be reliably detected in a diverse range of biospecimens, encompassing both conventional tumor biopsy samples and liquid biopsy samples (such as blood and urine). (**B**) Therapeutic potential of hypoxic ncRNAs. Inhibitors effectively modulate the expression and function of HIF-1α, while RNAi and ASO therapies underscore the precise targeting prowess of ncRNAs, specifically in the cytoplasm and nucleus, respectively.

Numerous hypoxia-related lncRNAs and circRNAs have been reported to be dysregulated in various cancer types. Additionally, they are highly specific and can be easily detected in tissues, serum, plasma, urine, and saliva. Therefore, hypoxia-related lncRNAs and circRNAs hold great promise as diagnostic and prognostic biomarkers and therapeutic targets in various cancers (Figure 4).

### 5.1. Biomarker Potentials

Ongoing clinical trials are actively recruiting patients to ascertain the potential of ncRNAs as diagnostic and prognostic biomarkers across various cancer types. Clinical research focusing on hypoxia-related lncRNAs and circRNAs offers a promising avenue for future exploration (Table 3). Thus, hypoxia-related lncRNAs and circRNAs have emerged as potential biomarkers for the diagnosis and prognosis of cancer (Figure 4A). A clinical study (NCT03469544) is currently investigating the potential of HOTAIR as a biomarker for thyroid cancer. Notably, HOTAIR, one of the most extensively investigated lncRNA, exhibits high specificity for RCC, and there is a positive correlation between its expression and the TNM stage [153]. Additionally, a notable elevation of HOTAIR levels was observed in CRC tissues relative to adjacent normal tissues (*p* = 0.00099), and survival analysis indicated that patients exhibiting a high level of HOTAIR had a poorer prognosis in CRC (*p* = 0.032) [154]. Another clinical trial (NCT04729855) is actively recruiting participants to examine the relationship between HOTTIP and CRC. In glioma patients, high HOTTIP expression was linked to poor overall survival, according to Kaplan–Meier survival analysis (*p* = 0.032) [76]. Additionally, ROC (receiver operating characteristics) analysis demonstrated that the diagnostic performance of the combination of circ-CDYL, HDGF, and HIF1AN for early-stage HCC (area under the curve [AUC] = 0.73) surpassed that of the traditional biomarker α-fetoprotein (AFP) (AUC = 0.59) [119]. In addition to these ncRNAs described above, several other hypoxia-related lncRNAs and circRNAs, including PVT1, HITT, BX111, circTDRD3, and circDENND4C, have shown promise as diagnostic and prognostic biomarkers in various cancers [63,65,80,106,121]. Notably, circDENND4C has been extensively evaluated in numerous independent studies as a prognostic biomarker for BC [148].

Clinically, liquid biopsy is receiving substantial attention owing to its less invasive nature relative to conventional tissue biopsy and its feasibility for real-time disease monitoring. Hypoxia-related circ101491, circ-133, and circHIF1A are overexpressed in peripheral blood plasma in a variety of malignancies, and this dysregulation correlates with a poor prognosis [109,125,138]. A clinical trial (NCT04584996) is investigating the expression of candidate circRNAs in tissue, blood, bile, and biopsy samples from patients with pancreaticobiliary cancer. Altogether, hypoxia-related lncRNAs and circRNAs can be used as biomarkers to improve the accuracy of cancer diagnosis and prognosis.

### 5.2. Therapeutic Potential

Low oxygen concentration, or hypoxia, is an important factor driving tumor aggressiveness. Clinical trials are underway to address hypoxia in cancer with investigational drugs (Figure 4B). In particular, a clinical study (NCT02564614) is examining the role of the *HIF-1α* mRNA antagonist RO7070179 in adult patients with liver cancer. Additionally, a phase 3 clinical trial (NCT04195750) is investigating the efficacy of the HIF-2α inhibitor MK-6482 in advanced renal clear cell carcinoma. Targeting HIF-1α and HIF-2α represents a promising therapeutic strategy for cancer. However, the emergence of resistance is inevitable. For instance, prolonged exposure to PT2399, a selective inhibitor of HIF-2, leads to the emergence of resistance associated with elevated tumor vascularity and increased levels of VEGF [93]. Therefore, developing novel and more effective therapeutic approaches is necessary to enhance treatment outcomes. Targeting the downstream effectors of the HIF signaling pathway is another promising approach to counteracting the hypoxia-induced invasiveness of cancer. The association of hypoxia-related lncRNAs and circRNAs with the onset and progression of cancer highlights their potential as therapeutic targets for cancer. Therefore, specific targeting of lncRNAs and circRNAs in the hypoxic TME represents a promising therapeutic strategy for complex and diverse cancer types.

Effective gene overexpression or knockdown techniques may facilitate the targeting of hypoxia-related lncRNAs and circRNAs for therapeutic purposes (Figure 4B). At present, RNA-based therapies primarily involve the use of RNA interference (RNAi) and antisense oligonucleotides (ASOs) to target specific regions and RNA molecules. Studies using mouse models have shown that targeting oncogenic hypoxia-related lncRNAs and circRNAs with ASOs and RNAi can effectively inhibit tumor growth. For instance, shRNAs can effectively target DANCR, a recently identified oncogenic lncRNA in NPC, to suppress tumor metastasis in nude mouse models [81]. Similarly, intravenous administration of shRNAs targeting circ-0001875 and circWSB1 can inhibit tumor proliferation in nude mouse models [108,111]. Various hypoxia-related circRNAs have been shown to regulate radiosensitivity and chemotherapy resistance in cancer. For instance, targeting cZNF292 with shRNAs enhances the radiosensitivity of HCC cells under hypoxic conditions in mice. Notably, the combination of radiotherapy and cZNF292 knockdown can reduce tumor volume in mice more effectively than radiotherapy or cZNF292 knockdown alone [118]. Targeting hypoxia-related circZNF91 with siRNAs can enhance GEM sensitivity in mice, whereas exosomal circZNF91 can transmit GEM resistance from hypoxic pancreatic cancer cells to normoxic pancreatic cancer cells [129]. Recently, a study in mice with Angelman syndrome showed that ASOs can be used to target lncRNAs [155]. The combination of LUCAT1 knockdown via ASO and oxaliplatin is reported to be more effective than oxaliplatin monotherapy in treating CRC in mice [66]. Conversely, the overexpression of suppressive hypoxia-related ncRNAs (e.g., lncRNA-LET and circEPHB4) has been shown to diminish tumor size and reduce metastases in nude mouse models [50,120]. Given their pivotal functional roles, hypoxia-related lncRNAs and circRNAs serve as promising therapeutic targets for cancer.

To date, several anti-hypoxia drugs based on HIF-1α, such as topotecan, and HIF-2α inhibitors, such as PT2399, have been clinically used for the treatment of cancer patients. Although some progress has been made in research on the role of hypoxia-related lncRNAs and circRNAs in cancer, clinical strategies for targeting these molecules are lacking at present. Therefore, large-scale, multi-center prospective cohort studies are warranted to validate the clinical utility of hypoxia-related lncRNAs and circRNAs in the development of new anti-cancer therapies for improving patient outcomes.

## 6. Concluding Remarks and Perspectives

The high death rate of cancer is due to its quick progression and lack of early diagnostic indicators. Consequently, there is an urgent imperative to identify novel tumor markers and therapeutic targets that can facilitate early diagnosis, accurate prognosis, and effective treatment. The hypoxia-related lncRNAs and circRNAs altered expression in the tumor specimens, and the easy detection of them in bodily fluids makes them attractive candidates for non-invasive liquid biopsy approaches. Moreover, these hypoxia-related ncRNAs have been shown to exert substantial effects in cancer hallmarks, including tumor metabolism, immune escape, tumor angiogenesis, migration/invasion, apoptosis, and growth/proliferation. Therefore, hypoxia-related lncRNAs and circRNAs have the potential to be used as biomarkers or therapeutic targets in solid tumors.

Although substantial progress has been made in examining the functions of lncRNAs and circRNAs in the hypoxic TME, certain unaddressed challenges and limitations warrant further investigation. In addition, the following questions remain unresolved: (i) When targeting hypoxia-related lncRNAs and circRNAs in cancer, how can we prevent their impact on essential gene expression in normal tissues? The identification of target ligands with high affinity and stability may help mitigate potential adverse effects and toxicity. Moreover, the refinement of delivery systems specifically engineered for the precise targeting of circRNA is paramount. (ii) Which factors contribute to the significant differences in hypoxic responses observed between cell lines and animal models? To date, most studies have predominantly focused on two-dimensional cell culture models; therefore, future studies should involve the use of three-dimensional tumor organ models that incorporate the TME [156]. (iii) Does the combination of hypoxia-related lncRNA- or circRNA-targeting therapies with other anti-tumor strategies yield superior therapeutic effects? In a recent study, the combination of ASO-mediated lncRNA downregulation with conventional chemotherapeutic agents demonstrated higher efficacy than chemotherapeutic agents alone in the treatment of CRC [66,118]. Therefore, the combination of hypoxia-related lncRNA- or circRNA-targeting therapies with other anti-cancer treatments holds substantial promise for enhancing treatment efficacy. (iv) Utilizing hypoxia-associated lncRNAs and circRNAs could be converted into RNA vaccines for tumor management in clinical settings. The advent of mRNA as a secure and efficacious platform in the race to develop a COVID-19 vaccine has imparted a swift understanding of the merits and potential risks associated with mRNA–LNP (a representative lipid nanoparticle) technology across the globe [157]. Notably, circRNAs, characterized by their substantial stability and attainable high translation efficiencies, have garnered considerable attention [158].

Notably, a gap exists between basic research findings and the clinical application of hypoxia-related lncRNAs and circRNAs as diagnostic, prognostic, and therapeutic biomarkers. Therefore, the therapeutic value and safety of hypoxia-related lncRNAs or circRNAs should be extensively investigated in future clinical studies with a large sample size.

## Figures and Tables

**Table 1:** Summary of hypoxia-related lncRNAs in cancers.

Hypoxia-Related lncRNAs	Expression	Interacting Partner	Target Genes/Pathways	Functions	Cancer Type	Reference
DACT3-AS1	↑	HDAC2/FOXA3	FOXA3	Facilitates HCC cell metastasis is achieved by enhancing FOXA3 deacetylation through promoting the interaction between HDAC2 and FOXA3	HCC	[47]
ALKBH3-AS1	↑		ALKBH3	Facilitates the proliferation and invasion of HCC cells by enhancing the stability of ALKBH3 mRNA	HCC	[48]
AC115619	↓		WTAP	Inhibits HCC cell metastasis by encoding a micropeptide that suppresses m6A modification	HCC	[49]
lncRNA LET	↓	NF90	HIF-1α	Inhibits HCC metastasis by decreasing the mRNA expression of HIF-1α through enhanced NF90 degradation.	HCC	[50]
FRMD6-AS1	↑	SENP1	HIF-1α	Promotes HCC cell metastasis is achieved by enhancing the protease activity of SENP1 to regulate SUMOylation of HIF-1α	HCC	[51]
Linc-RoR	↑	miR-145	HIF-1α	Facilitates HCC cell proliferation by the miR-145/HIF-1α axis	HCC	[52]
EIF3J-AS1	↑	miR-122-5p	CTNND2	Promotes HCC cell proliferation and invasion by the miR-122-5p/CTNND2 axis	HCC	[53]
HMMR-AS1	↑	miR-147	ARID3A	Promotes HCC cell proliferation and influences macrophage polarization via the miR-147/ARID3A axis	HCC	[54]
lncRNA PMAN	↑	ELAVL1	SLC7A11	Facilitates GC cell metastasis by promoting the cytoplasmic localization of ELAVL1 to increase the stability of SLC7A11 mRNA	GC	[55]
BC005927	↑		EPHB4	Promotes GC cell metastasis by upregulating EPHB4	GC	[56]
lncRNA AK058003	↑		SNCG	Promotes GC metastasis by regulating SNGG via DNA demethylation	GC	[57]
PVT1	↑	miR-186	HIF-1α	Facilitates GC cell invasion by the miR-186/HIF-1α axis	GC	[58]
ZEB2-AS1	↑	miR-143-5p	HIF-1α	Promotes GC cell proliferation and invasion by the miR-143-5p/HIF-1α axis	GC	[59]
lncRNA CF129	↓	p53	FOXC2	Inhibits pancreatic cell proliferation and invasion by inducing the MKRN1-mediated ubiquitin-dependent degradation of p53	Pancreatic cancer	[60]
NORAD	↑	hsa-miR-125a-3p	RhoA	Facilitates pancreatic cancer cell metastasis by the miR-125a-3/RhoA axis	Pancreatic cancer	[61]
lncRNA MTA2TR	↑	ATF3	MTA2	Facilitates proliferation and invasion by recruiting ATF3 to the MTA2 promoter region to activate the transcription of *MTA2*	Pancreatic cancer	[62]
lncRNA BX111	↑	YB1	ZEB1	Promotes pancreatic cancer cell metastasis by recruiting YB1 to the ZEB1 promoter region to activate the transcription of *ZEB1*	Pancreatic cancer	[63]
ENST0000480739	↓		OS-9/HIF-1α	Inhibits pancreatic cancer cell invasion by suppressing HIF-1α expression	Pancreatic cancer	[64]
HITT	↓		YBX1	Suppresses CRC angiogenesis and tumor growth by binding with YB-1 to downregulate HIF-1α	CRC	[65]
LUCAT1	↑	PTBP1		Facilitates CRC cell viability and chemotherapy response by modifying the alternative splicing of DNA damage-related genes through interaction with PTBP1	CRC	[66]
STEAP3-AS1	↑	STEAP3	Wnt/β-catenin signaling	Promotes CRC cell proliferation and metastasis by preventing m6A-mediated degradation of STEAP3 mRNA	CRC	[67]
LVBU	↑	miR-10a/miR-34c	BCL6	Facilitates CRC cell proliferation by sponging miR-10a/miR-34c to enhance BCL6 expression	CRC	[68]
ELFN1-AS1	↑	miR-191-5p	TRIM14	Promotes CRC cell proliferation and invasion by sponging miR-191-5p to increase TRIM14 expression	CRC	[69]
DLEU1	↑	CKAP2	ERK/STAT3	Promotes BC cell metastasis by interacting with CKAP2 to activate the ERK and STAT3 signaling pathways	BC	[70]
LINC00649	↑	NF90/NF45	HIF-1α	Facilitates BC cell metastasis by maintaining the stability of HIF-1α through the NF90/NF45 complex	BC	[71]
EFNA3	↑	miRNA-210	EFNA3	Facilitates BC cell metastasis by the miR-210/EFNA3 axis	BC	[72]
NDRG1-OT1	↑	miR-875-3p		Facilitates BC cell metastasis via sponging miR-875-3p	BC	[73]
HIF1A-AS2	↑	HMGA1	DHX9/IGF2BP2	Promotes the expression of HMGA1 by interacting with DHX9 and IGF2BP2 mRNA binding complexes	Glioma	[74]
lncRNA H19	↑	miR-181d		Promotes glioma cell migration and invasion by acting as a ceRNA of miR-181d to increase β-catenin expression	Glioma	[75]
HOTTIP	↑	miR-101	ZEB1	Facilitates epithelial-mesenchymal transition through the miR-101/ZEB1 axis	Glioma	[76]
lncRNA SARCC	↑	AR	HIF-2α/C-MYC	Inhibits proliferation of VHL-mutant cells yet promotes proliferation of VHL-normal cells via modulating the AR/HIF-2α/C-MYC axis	RCC	[77]
RP11-367G18.1 V2	↑		H4K16Ac	Facilitates RCC cell metastasis by promoting acetylation of H4K16Ac	RCC	[78]
HOTAIR	↑	miR-217	HIF-1α/AXL	Facilitates RCC cell metastasis and EMT by regulating miR-217/HIF-1α/AXL signaling pathway	RCC	[79]
PVT1	↑	KAT2A	HIF-1α	Promotes nasopharyngeal carcinoma cell proliferation by activating KAT2A to increase the stability of HIF-1α	NPC	[80]
DANCR	↑	NF90/NF45	HIF-1α	Improves HIF-1α mRNA stability through interacting with the NF90/NF45 complex	NPC	[81]
NEAT1	↑	miR-101-3p	OX9/Wnt/β-Catenin	Promotes NSCLC progression via the miR-101-3p/SOX9/Wnt/β-Catenin signal pathway	NSCLC	[82]
LHFPL3-AS2	↓	SFPQ	TXNIP	Suppresses NSCLC cell metastasis by interacting with SFPQ to regulate TXNIP expression	NSCLC	[83]
MIR17HG	↑	miR-155-5p	HIF-1α	Promotes retinoblastoma cell proliferation and invasion by the miR-155-5p/HIF-1α axis	Retinoblastoma	[84]
HIFCAR	↑	HIF-1α/p300		Facilitates the recruitment of HIF-1α and the p300 cofactor to the target gene promoters	Oral carcinoma	[85]
lincRNA-p21	↑	VHL/HIF-1α		Enhances glycolysis by inhibiting the VHL-mediated ubiquitination of HIF-1α	Cervical cancer	[86]
DARS-AS1	↑	METTL3/METTL14	DARS	Promotes cervical cancer cell autophagy by facilitating the translation of DARS through METTL3- and METTL14-mediated m6A modification	Cervical cancer	[87]
DARS-AS1	↑	RNF147	RBM39	Facilitates multiple myeloma cell proliferation and apoptosis by regulating RBM39 stability through inhibiting its interaction with RNF147	Multiple myeloma	[88]
WT1-AS	↑	H3K4/H3K9	WT-1	Promotes myeloid leukemia cell proliferation by modulating the methylation of H3K4 and H3K9 to facilitate WT-1 upregulation	Myeloid leukemia	[89]
MEG3	↑	DNMT3a/DNMT3b/MBD1	TIMP2	Facilitates pheochromocytoma cell proliferation by recruiting DNMT3a, DNMT3b, and MBD1 to accelerate TIMP2 promoter methylation	Pheochromocytoma	[90]

Abbreviations: BC, breast cancer; CRC, colorectal cancer; GC, gastric cancer; HCC, hepatocellular carcinoma; NPC, nasopharyngeal carcinoma; NSCLC, non-small cell lung cancer; RCC, renal cell carcinoma; ↑, upregulate; ↓, downregulate.

**Table 2:** Summary of hypoxia-related circRNAs in cancers.

Hypoxia-Related circRNAs	Expression	Interacting Partner	Target Genes/Pathways	Functions	Cancers	Reference
CircRNF20	↑	miR-487a	HIF-1α/HK2	Contributes to BC cell proliferation and aerobic glycolysis	BC	[103]
CircZFR	↑	miR-578	HIF-1α	Facilitates BC progression	BC	[104]
CircRBM33	↑	miR-542-3p	HIF-1α	Facilitates hypoxia-induced glycolysis	BC	[105]
CircDENND4C	↑	miR-200b/miR-200c		Promotes BC cell proliferation under hypoxic conditions	BC	[106]
CircTBC1D14	↑	FUS	PRMT1	Maintains cellular homeostasis and promotes tumor progression	BC	[107]
CircWSB1	↑		USP10	Promotes the proliferation of BC cells	BC	[108]
CircHIF-1α	↑		NFIB/FUS	Facilitates the development and metastasis of BC	BC	[109]
CircPFKFB4	↑	DDB1/DDB2	CRL4DDB2/p27	Facilitates BC progression	BC	[110]
circ-0001875	↑	miR-31-5p	SP1	Facilitates the progression of NSCLC	NSCLC	[111]
CircPIP5K1A	↑	miR-600	HIF-1α	Facilitates tumor proliferation and metastasis	NSCLC	[112]
hsa-circ-0000211	↑	miR-622	HIF-1α	Promotes cancer cell migration and invasion	LUAD	[113]
Circ0061140	↑	miR-653	HK2	Accelerates hypoxia-induced glycolysis, migration, and invasion in LUAD	LUAD	[114]
CircRNA CCDC66	↑			Advances drug resistance and EMT	LUAD	[115]
CircMAT2B	↑	miR-338-3p	PKM2	Facilitates HCC progression by enhancing glycolysis	HCC	[116]
CircPRDM4	↑		HIF-1α	Facilitates immune escape of HCC cells	HCC	[117]
cZNF292	↑	miR-23b-3p	Wnt/β-catenin	Promotes the hypoxia-induced proliferation of human hepatoma SMMC7721 cells, radioresistance, and vasculogenic mimicry	HCC	[118]
CircCDYL	↑	miR-328-3p	HIF-1AN	Promotes stem-like characteristics and tumor development	HCC	[119]
Circ-EPHB4	↓		HIF-1α/PI3K-AKT	Inhibits the development, growth, and metastasis of HCC	HCC	[120]
CircTDRD3	↑	miR-1231	HIF-1α	Facilitates the growth, migration, and metastasis of CRC cells	CRC	[121]
Circ_0006508	↑	miR-1272	HIF-1α	Promotes the viability and Warburg effect of CRC in vitro	CRC	[122]
Circ-Erbin	↑	miR-125a-5p/miR-138-5p	4EBP-1	Promotes CRC cell proliferation and metastasis	CRC	[123]
CircEXOC6B	↓		RRAGB	Inhibits the development of CRC cells and increases 5-fluorouracil-induced apoptosis	CRC	[124]
Circ-133	↑	miR-133a	GEF-H1/RhoA	Facilitates metastasis	CRC	[125]
Circ_0000977	↑	miR-153	HIF1/ADAM10	Modulates the HIF-1α-mediated immune evasion of pancreatic cancer cells in vitro	Pancreatic cancer	[126]
CircRNF13	↑	miR-654-3p	PDK3	Promotes the malignant progression of pancreatic cancer	Pancreatic cancer	[127]
CircPDK1	↑	miR-628-3p/BPTF	c-myc	Facilitates the proliferation, migration, and glycolysis of pancreatic cancer cells	Pancreatic cancer	[128]
CircZNF91	↑		SIRT1 and HIF-1α	Promotes the resistance of pancreatic cancer cells to GEM	Pancreatic cancer	[129]
ebv-circLMP2A	↑	KHSRP	HIF-1α	Promotes angiogenesis in EBV-associated GC	GC	[130]
CircDNMT1	↑	miR-576-3p	HIF-1α	Promotes the malignant progression of GC	GC	[131]
CircC6orf132	↑	miR-873-5p	PRKAA1	Promotes GC cell proliferation, migration, invasion, and glycolysis in a hypoxic environment in vitro and in vivo	GC	[132]
CircCCDC134	↑	miR-503-5p	HIF-1α	Stimulates HIF-1α transcription and facilitates the growth and metastasis of cervical cancer	Cervical cancer	[133]
Circ_0004543	↑	miR-217	HIF-1α	Aggravates cervical cancer development	Cervical cancer	[134]
Circ-HIPK3	↑	miR-338-3p	HIF-1α	Facilitates the EMT of cervical cancer cells	Cervical cancer	[135]
CircDENND2A	↑	miR-625-5p		Facilitates the aggressiveness of glioma	Glioma	[136]
CircADAMTS6	↑		ANXA2	Promotes cell proliferation and inhibits apoptosis, accelerating the development of glioblastoma	Glioma	[137]
Circ101491	↑	miR-125b-5p	EDN1	Promotes the viability, invasiveness, and migration of glioma cells	Glioma	[138]
CircBCAR3	↑	miR-27A-3p	TnPO1	Facilitates the migration and ferroptosis of ESCC	ESCC	[139]
CircPUM1	↑		UQCRC2	Inhibits ESCC cell pyroptosis, thereby promoting the proliferation of ESCC cells both in vivo and in vitro	ESCC	[140]
CircELP3	↑			Promotes the development of bladder cancer and cisplatin resistance	Bladder cancer	[141]
CircCYP51A1	↑	miR-490-3p	KLF12	Mediates cancer cell proliferation, migration, invasion, and glycolysis	Osteosarcoma	[142]
CircSTX6	↑	miR-449b-5p	HIF-1α	Promotes tumor proliferation and metastasis both in vitro and in vivo	PDAC	[143]
CDR1as	↓	miR-135b-5p	HIF-1AN	Inhibits the development of ovarian cancer in vitro	Ovarian cancer	[144]
CircRTN4IP1	↑	miR-541-5p	HIF-1α	Facilitates tumor formation in vivo	ICC	[145]

Abbreviations: BC, breast cancer; CRC, colorectal cancer; ESCC, esophageal squamous cell carcinoma; GC, gastric cancer; HCC, hepatocellular carcinoma; ICC, intrahepatic cholangiocarcinoma; LUAD, lung adenocarcinoma; NSCLC, non-small cell lung cancer; PDAC, pancreatic ductal adenocarcinoma; ↑, upregulate; ↓, downregulate.

**Table 3:** Examples of preclinical and clinical trials exploring the application of hypoxia-related ncRNAs in cancers.

NcRNAs	Expression	Cancer Type	Source	Sample Size	Sample Grouping	Clinical Significance	Reference
lncRNA HOTTIP	↑	Glioma	Tissues	60|56	Metastatic glioma patients/non-metastatic glioma patients	Associated with metastatic progression (*p* < 0.0001)	[76]
lncRNA PVT1	↑	NPC	Tissues	10|10	NPC patients/healthy donors	Associated with diagnosis and prognosis (*p* < 0.001)	[80]
lncRNA HITT	↓	CRC	Tissues	46|46	CRC patient/matched adjacent controls	Associated with diagnosis and prognosis (*p* < 0.01)	[65]
lncRNA BX111	↑	Pancreatic cancer	Tissues	17|31	Resected/non-resected	Associated with metastatic progression (*p* < 0.01)	[63]
circTDRD3	↑	CRC	Tissues	106|106/55|55	CRC patients/matched adjacent controls	Associated with tumor size, TNM stage, and lymph node invasion (*p* < 0.05)	[121]
circDENND4C	↑	BC	Tissues	30|30	BC patients/matched adjacent controls	Associated with tumor size (*p* < 0.0001)	[106]
circ101491	↑	Glioma	Blood sample	20|20	Cancer patients/healthy donors	Associated with the degree of tumor differentiation and tumor TNM staging (*p* = 0.037 and *p* = 0.009, respectively)	[138]
circ-133	↑	CRC	Blood sample	10|10	Cancer patients/healthy donors	Associated with TNM staging (*p* < 0.01)	[125]
circHIF1A	↑	BC	Blood sample	101|84	CRC patients/matched adjacent controls	Associated with overall survival (OS) (*p* = 0.025)	[109]
lncRNA HOTAIR	↑	Thyroid Cancer	Blood sample	90 participants	Cancer patients/normal healthy subjects	Associated with diagnosis	NCT03469544
lncRNA HOTTIP	↑	CRC	Blood sample	60 participants	Cancer patients/normal healthy subjects	Associated with diagnosis	NCT04729855

Abbreviations: BC, breast cancer; CRC, colorectal cancer; NPC, nasopharyngeal carcinoma; OS, overall survival; ↑, upregulate; ↓, downregulate.

## Data Availability

Not applicable.
